# Comparative Study on the Phytochemical Composition and Antioxidant Activity of Grecian Juniper (*Juniperus excelsa* M. Bieb) Unripe and Ripe Galbuli

**DOI:** 10.3390/plants9091207

**Published:** 2020-09-15

**Authors:** Stanko Stankov, Hafize Fidan, Zhana Petkova, Magdalena Stoyanova, Nadezhda Petkova, Albena Stoyanova, Ivanka Semerdjieva, Tzenka Radoukova, Valtcho D. Zheljazkov

**Affiliations:** 1 Department of Nutrition and Tourism, University of Food Technologies, 26 Maritza, 4002 Plovdiv, Bulgaria; docstankov@gmail.com (S.S.); hfidan@abv.bg (H.F.); 2 Department of Chemical Technology, University of Plovdiv Paisii Hilendarski, 24 Tzar Asen, 4000 Plovdiv, Bulgaria; zhanapetkova@uni-plovdiv.net; 3 Department of Analytical Chemistry and Physicochemistry, University of Food Technologies, 26 Maritza, 4002 Plovdiv, Bulgaria; magdalena.stoianova@abv.bg; 4 Department of Organic Chemistry and Inorganic Chemistry, University of Food Technologies, 26 Maritza, 4002 Plovdiv, Bulgaria; petkovanadejda@abv.bg; 5 Department of Technology of Fats, Essential Oils, Perfumery and Cosmetics, University of Food Technologies, 26 Maritza, 4002 Plovdiv, Bulgaria; aastst@abv.bg; 6 Department of Botany and Agrometeorology, Agricultural University, 12 Mendleev12, 4000 Plovdiv, Bulgaria; v_semerdjieva@abv.bg; 7 Department of Botany and Methods of Biology Teaching, University of Plovdiv Paisii Hilendarski, 24 Tzar Asen, 4000 Plovdiv, Bulgaria; kiprei@abv.bg; 8 Crop and Soil Science Department, Oregon State University, 3050 SW Campus Way, 109 Crop Science Building, Corvallis, OR 97331, USA

**Keywords:** Grecian juniper, galbuli, maturity stage, chemical composition, ethanol extracts

## Abstract

Grecian juniper (*Juniperus excelsa* M. Bieb.) is an evergreen tree and a rare plant found in very few locations in southern Bulgaria. The aim of this study was to evaluate the phytochemical content and antioxidant potential of *J. excelsa* unripe and ripe galbuli from three different locations in Bulgaria. The essential oil content ranged between 1.9% and 5.1%, while the lipid fraction yield was between 4.5% and 9.1%. The content of total chlorophyll was 185.4–273.4 μg/g dw. The total carotenoid content ranged between 41.7 and 50.4 μg/g dw of ripe galbuli, and protein content was between 13.6% and 16.4%. Histidine (5.5 and 8.0 mg/g content range) and lysine (4.0 and 6.1 mg/g) were the major essential amino acids. The antioxidant potential of the 95% and 70% ethanol extracts was analyzed using four different methods. A positive correlation between the antioxidant potential and phenolic content of the galbuli was found. The results obtained in this study demonstrated the differences in phytochemical composition and antioxidant capacity of *J. excelsa* galbuli as a function of maturity stage and collection locality.

## 1. Introduction

The genus *Juniperus* (Cupressaceae) contains more than 60 species, widespread, mainly in the northern hemisphere including North America, Europe, and Asia [1]. *Juniperus excelsa* is an evergreen tree species up to a height of 15 m, with medicinal and landscaping importance. The galbuli (cones) are spherical with a diameter of 7 to 12 mm and are covered with a grayish-gray coating [2]. The habitats of *J. excelsa* form endemic juniper forests, and they are very rare in the European countries. The species is included in the IUCN Red list [3] and Red Data Book of the Republic of Bulgaria under the “Critically Endangered” category [4]. In Bulgaria, *J. excelsa* is a rare plant species protected by the Biological Diversity Law in Bulgaria [4]. The locations of *J. excelsa* populations in Bulgaria represent the northernmost areal of distribution of this species [4]. In Bulgaria, the plant grows in places with Mediterranean and temperate continental climate on the steep slopes of deep gorges in the Western Rhodopes (the reserve “Izgoryaloto Gyune”), and more commonly occurs in the valley of the Struma river (the reserve “Tisata”). The latter reserve includes thousands of *J. excelsa* trees, making it the most representative and numerous population of this species in Bulgaria.

Because of its phytochemical composition, *Juniperus* species are used in folk medicine; they are widely used in the treatment of various diseases such as cough, cold, hemorrhoids, fungal infections, etc. [5,6].

Juniper galbuli has been used for the treatment of cardiac and nervous disorders; parasitic diseases; diuretic complications provoked by eating disorders in several countries such as Bosnia and Herzegovina, Lebanon, Iran, and Turkey [7]. Some previous reports have shown diversities in the chemical composition of the *J. excelsa* galbuli essential oils from different parts of the world [8,9,10,11,12]. The diversities in the quantity and quality of the volatile oils are the function of genetic and nongenetic variables such as climatic, edaphic conditions, the season and time of the harvest, and even the duration of the sunlight exposure [1].

*Juniperus excelsa* galbuli are rich in phenolic compounds such as gallic acid, cinnamic acid, vanillic acid, hydroxybenzoic acid, sinapic acid, ellagic acid, myrcetin, and hesperidin [13] and demonstrated various biological effects such as antimicrobial [11,12,14,15], antifungal [16], antioxidant [17,18,19,20], anti-inflammatory [21], anticancer [22], antiviral, and cytotoxic activities [11]. Phytochemical analysis of various anatomical parts of the genus *Juniperus* showed the presence of sterols, flavonoids, lignans [23,24,25], polysaccharides [26], some aromatic compounds, and fatty acids [27].

The use of various synthetic antioxidants in food production leads to a deterioration of the taste and biologically active qualities of food, and may lead to conditions suitable for the occurrence of food allergies. These negative effects draw the researchers’ attention to the exploration of alternative sources of reactive components that prevent the occurrence of oxidative processes, which have negative effects on cellular metabolism. Knowledge of the antioxidant properties of many plant species allows their usage as a means of preserving food quality by slowing down or preventing lipid oxidation processes [28,29].

However, there is no information on the composition and the antioxidant activity of unripe and ripe galbuli of *J. excelsa*. Therefore, the goal of this study was to investigate the phytochemical composition and to assess the content of phenolic compounds, flavonoids, as well as the antioxidant capacity of *J. excelsa* galbuli extracts. The working hypothesis was that the phytochemical composition, phenolic compounds, and antioxidant activity of *J. excelsa* galbuli will depend on their maturity phase (unripe and ripe) and collection location.

## 2. Results

### 2.1. Proximate Composition

The moisture, total crude protein, chlorophyll *a*, chlorophyll *b*, total chlorophyll, total carotenoids, essential oil yield, and lipid fraction yield of *J. excelsa* ripe and unripe galbuli derived from different locations in Bulgaria are shown in Table 1.

The protein content was the highest (16.4%) in the unripe *J. excelsa* galbuli samples collected from location 2. The protein content in unripe galbuli (locations 1 and 2) was higher than that of ripe galbuli from the same areas. The ripe galbuli (location 3) had a higher level of protein (15.4%) than the unripe ones, as the values were comparable to those of the unripe galbuli from locations 1 and 2. The differences in the amount of the protein fraction in the unripe and ripe galbuli of *J. excelsa* may be due to the difference in the reported moisture of the samples, as there was a relationship between the amount of moisture and the proteins contained in their composition.

The content of total chlorophyll was the highest in the ripe sample from location 3 (273.4 μg/g dw), as the chlorophyll a and chlorophyll b were obtained as 193.1 and 80.3 μg/g dw, respectively. The lowest concentrations of total chlorophyll were found in the unripe galbuli sample of the same location. The total carotenoid content ranged between 41.7 (ripe galbuli from location 2) and 50.4 μg/g dw (ripe galbuli from location 3).

The ripe galbuli of *J. excelsa* (location 1) showed a maximum essential oil yield of 5.1%, followed by the ripe galbuli sample from locations 2 and 3 (2.6% and 2.5%, respectively) (Table 1). Overall, the ripe galbuli were characterized by a higher essential oil content than the unripe samples (Table 1).

The lipid fraction content (ranging between 4.5% and 9.1%) was the highest for the ripe galbuli sample of *J. excelsa* from all locations.

### 2.2. Composition of the Lipid Fraction

The fatty acid composition of the lipid fractions from *J. excelsa* unripe and ripe galbuli is presented in Table 2.

The fatty acid composition of the samples was heterogeneous, and the results did not show any dependencies between the individual samples. The amount of linoleic fatty acid (26.2%) was the highest in the unripe galbuli from location 1 compared to the other samples. The content of saturated fatty acids (palmitic fatty acid) in the unripe galbuli sample (location 2) was the highest (41.3%), compared to the other samples. Palmitic acid was the major fatty acid in the lipids from almost all galbuli, apart from those from location 3. The content of oleic acid was the lowest in unripe galbuli from location 2 (8.4%), and highest in the ripe sample from location 3 (32.6%). The quantity of stearic acid ranged between 4.5% and 8.4%. A small amount of linolenic acid (1.3–4.8%) was also found in all samples. Eicosatrienoic (1.7–8.8%) and lignoceric (1.8–6.4%) acids were found in all samples as well.

Table 3 represents the content of unsaponifiable matter, sterols, and tocopherols of *J. excelsa* unripe and ripe galbuli from different areas of Bulgaria.

The highest concentrations of unsaponifiable matter were obtained for the lipid fraction of ripe galbuli (13.5%) from location 3, followed by the ripe (11.4%) and unripe (11.6%) galbuli samples from location 2.

The concentration of sterols was also the highest in the lipid fraction from the ripe galbuli from locations 2 and 3 (0.3%). The total sterols in the galbuli from location 2 increased with ripening; however, a decrease in total sterols in the lipid fraction of the galbuli from location 1 was observed. The total tocopherol composition of the lipid fraction of the ripe galbuli from location 1 was the highest (1894 mg/kg), while the lowest concentrations (721 mg/kg) were found in the unripe galbuli from location 2. Overall, the total amount of tocopherols increased with the ripening of the galbuli.

Table 4 shows the sterols and tocopherols in the lipid fraction of *J. excelsa* and had the highest concentration of *β*-sitosterol (91.7%) in the ripe galbuli from location 1.

The main representative of the sterols was *β*-sitosterol, whose lowest concentrations were obtained for the lipid fraction of the ripe galbuli from location 3. The same sample had the highest concentration of campesterol. Apparently, the concentration of *β*-sitosterol slightly decreased during the ripening of the galbuli from location 2, while in the galbuli from location 1, the concentration of *β*-sitosterol significantly increased with ripening. A considerable increase with ripening was also observed in the quantity of Δ^7^–stigmasterol in the galbuli from location 2. *α*-Tocopherol and *α*-tocotrienol were identified in all the analyzed samples. On the other hand, *γ*-tocopherol was present in the lipids from ripe galbuli from locations 1 and 2; while β-tocopherol was identified only in ripe galbuli from location 2.

*α*-Tocopherol can be used as a natural source of preservative or synergist to antioxidants in the food industry or in the production of animal feed. The high antioxidant potential of tocopherols in ripe juniper galbuli may have a potential to be used in canning and marinating foods.

### 2.3. Amino Acid Composition of the Protein Fraction

The amino acid composition of the protein fraction of *J. excelsa* unripe and ripe galbuli from different locations is shown in Table 5. Histidine was the major essential amino acid (concentration range of 5.5–8.0 mg/g) in all tested samples, followed by lysine. Among the nonessential amino acids, asparagine (concentration range of 3.4–8.6 mg/g), alanine (concentration range of 4.6–7.1 mg/g), and glutamic acid (concentration range of 2.8–6.7 mg/g) were the amino acids with the highest concentrations in all tested samples. The results showed that the ripening of galbuli had a significant effect on amino acid composition.

### 2.4. Composition and Antioxidant Activity of Ethanol Extracts

The total polyphenols, flavonoid content, and antioxidant activity of 95% and 70% ethanol extracts of *J. excelsa* unripe and ripe galbuli from three different locations are presented in Table 6. The total polyphenolic and flavonoid content of 95% ethanol extract of galbuli were the highest in the unripe galbuli from location 3 (4.3 and 4.3 mg QE/g dw). The 70% ethanol extract of unripe galbuli from the same location 3 was characterized with the highest content, as 12.3 and 1.7 mg QE/g dw, respectively, and also showed the highest antioxidant potential by copper reduction assay 473.9 mM TE/g dw.

A significant correlation was found between the total phenols and the antioxidant activity by the DPPH, ABTS, FRAP and CUPRAC methods (Table 7).

## 3. Discussion

The use of plants as food and medicines has been known since ancient times. With global change related to the temperature increase and displacement of precipitation patterns during the seasons, the natural habitats of different plant species, as well as their phytochemical characteristics, are likely to change [30]. Plants respond to climate changes with phenotypic plasticity, variation in physiology, and reproduction capacity. As mentioned above, the habitats of *J. excelsa* form endemic juniper forests, and the species is a rare and protected plant. There are several reasons for the limited distribution of the *J. excelsa*: (1) very slow growth of the species; (2) the plants grow under extreme conditions (steep slopes of deep gorges, very high temperatures, frequently on very thin soil layer); (3) *J. excelsa* seeds have long dormancy, and they are characterized by low germination percentage and slow seedling growth [31]. The reserve “Izgoryaloto Gyune” (location 1) is located on the northern slopes of the Western Rhodopes, where the soil layer is almost absent and the climate is temperate continental, while the reserve “Tisata” (locations 2 and 3) is situated on two horsts belonging to Malashevska Mountain and South Pirin Mountain, where the climate is Mediterranean [32]. The predominant soil type is Chromic Luvisol [32]. Location 3 is positioned on the eastern slopes of the Pirin Mountain with a longer duration of sunlight exposure.

### 3.1. Moisture, Chlorophyll a and b, and Carotenoid Content

Our investigation shows that ripe and unripe galbuli of *J. excelsa* had different moisture contents. The phenological stage of galbuli determines their moisture. Apparently, a high level of moisture content in unripe galbuli was found because of higher water in cells protoplast. At location 3, an inverse relationship was reported in which the moisture of the ripe galbuli was slightly higher (32%) compared to that of the unripe samples (31.9%). The trees in location 3 are found on the southern slopes, and increased sunlight exposure is known to improve photosynthesis and increase the amount of chlorophyll *b* in ripe fruit (80.3 μg/g dw—location 3).

Our study shows that mature and immature galbuli from *J. excelsa* have different pigment composition of chlorophyll *a* and *b* and carotenoid. The higher levels of pigments in ripe galbuli probably depend on plastid development. An inverse relationship was reported at location 3, due to the more pronounced processes of photosynthesis and the stage of ripening, in which the galbuli may change the color of the outer wall but may retain the high protein content. Traditionally, changes in the content of chlorophylls (*a*/*b*) and carotenoids (photosynthetic capacity) in plants have been used as an indicator for abiotic stress [33]. The examination of pigmentations of plants is an important parameter for ecophysiologists and it may provide information on the availability of nitrogen, free carbohydrates, or water during plant ontogeny [34]. The photosynthetic parameters (chlorophylls and carotenoids) are related to other functions such as growth, reproduction and the phytochemical production, and structural defenses [35]. Furthermore, chlorophylls and carotenoids are common pigments, which give the color of fruit, vegetables, etc. Because of their colors and chemical features, they are also used as food additives [34,35,36]. Overall, the chlorophylls (*a*/*b*) and carotenoids of *J. excelsa* leaves have been reported; however, there was no comprehensive study of chlorophylls of *J. excelsa* galbuli. The total content of chlorophylls remained the same or increased during ripening of the galbuli from location 1 and 3, but slightly decreased in the ripe ones from location 2. A similar reduction in the total content of chlorophylls and carotenoids was observed during the development of flaxseed hulls by Herchi et al. [37].

### 3.2. Amino Acid Composition of the Protein Fraction

The *J. excelsa* is a seed producing plant that may form galbuli in 2–3 years. A morphological polymorphism exists in this species, and the number of seeds per galbuli appears to be the most variable characteristic [38]. Two or three seeds are formed in the galbuli, which can explain the differences in the protein and amino acid content between the samples. Furthermore, the lower degree of photosynthesis slows down the maturation processes, in which changes related to the breakdown of cell walls and protein composition may occur [39]. This confirms the differences obtained, in which higher levels of protein in unripe galbuli were reported (locations 1 and 2). Total protein content in *J. excelsa* galbuli from location 3 increased during ripening but slightly decreased in the galbuli from locations 1 and 2. The results are in agreement with those reported for other plants [40,41]. According to latter reports, this decrease could be explained by the protein breakdown after which the obtained amino acids were utilized in gluconeogenesis [40,41].

### 3.3. The Yield Essential Oil

The essential oil yield has been previously reported [8,9], while the information about the lipid fraction yield of juniper galbuli has been limited. The essential oil yield in this study was higher than those reported by Angioni et al. [14] for unripe and ripe galbuli collected from three other *Juniperus* species (*J. oxycedrus* L. ssp. *oxycedrus*, *J. phoenicea* ssp. *turbinata*, and *J. communis* ssp. *communis*). The latter authors obtained the highest essential oil yield from ripe galbuli from *J. phoenicea* ssp. *turbinata*. Al Hafi et al. [8] studied the chemical composition of the essential oils from berries, leaves, and twigs of *J. excelsa* growing wild in Lebanon and determined that the essential oil yield was highest in the galbuli (0.1–2.5%). The yield of *J. excelsa* galbuli essential oil obtained by hydrodistillation by Azzimonti et al. [9] for the essential oil obtained from the galbuli of Lebanese *J. excelsa* was 1.17%. The results from this study are in agreement with those reported previously by Goze et al. [10] for *J. excelsa*. subsp *excelsa* galbuli essential oil content (5.8% *v/w*) from Turkey. Topçu et al. [11] reported that the yield of *J. excelsa* galbuli essential oil was 3.2% (*v/w*).

### 3.4. Fatty Acid Composition of the Lipid Fraction

Lipids were the main constituents in the seed. The main fatty acids were palmitic, oleic, and linoleic. The low levels of the lipid fraction in the studied ripe galbuli were comparable with the data for other juniper species, e.g., *J. occidentalis* (16%) [42] and *J. phoenicea* (11%) [43]. It was stated that the lipid fraction of *J. drupacea* may vary depending on the altitude and age of the trees, as at lower altitude the lipid fraction was 5.5%, and at higher (above 1200 m), it was 3.8% [44].

Total lipid fraction increased during the ripening of the galbuli from location 1 but slightly decreased in the galbuli from location 2. A decline in the lipids during the development of culinary banana (different species) was previously reported [40,41]. In this study, the highest concentrations of linoleic acid were obtained for unripe and ripe *J. excelsa* galbuli from location 1. The linoleic acid content in this study was similar to that reported by other researchers [11,27]; however, the subject of these previous studies was only ripe galbuli of *Juniperus* species. High concentrations of oleic acid were found in ripe galbuli from locations 2 and 3, and these results are comparable to the data reported for galbuli of other juniper species [45,46]. The concentrations reported for linoleic acid of *J. oxycedrus* ripe galbuli by Ozkaya et al. [47] were similar to linoleic acid content of *J. excelsa* unripe and ripe galbuli from location 1 in this study.

Results from this study showed that the maturity did not affect the fatty acid composition of *J. excelsa* galbuli. Palmitic acid was the main fatty acid in the *J. excelsa* unripe galbuli from location 2. However, a tendency of decreasing the quantity of palmitic and linoleic acids and increasing the amount of stearic and oleic acids during ripening was observed in both lipid fractions of galbuli from locations 1 and 2. Factors influencing the fatty acid composition of the samples are climatic conditions, precipitation, and soil chemical, physical, and biological properties.

The highest total unsaturated fatty acid content was found in ripe galbuli from location 2 followed by the unripe galbuli obtained from the same study area. The ripe galbuli from location 3 had similar concentrations of unsaturated fatty acids compared with that of the unripe galbuli from location 1. The observed variation in fatty acid content and composition could be due to the different environmental conditions at the three collection sites. The ripe galbuli from location 2 had the highest polyunsaturated fatty acid content. The saturated fatty acid content also varied depending on the location.

### 3.5. Total Polyphenols, Flavonoids, and Antioxidant Activity

Many phytosterols are present in plants. *β*-Sitosterol, campesterol, and stigmasterol are among the main biosynthetic final products in the phytosterol pathway. Stigmasterol, known as one of the main phytosterols, has been the object of previous studies because of its significance as an anti-inflammatory, antioxidant, and antitumor agent [48]. In this study, the unripe galbuli from location 1 contained a higher percentage of stigmasterol than the other samples. *α*-Tocopherol was the major tocopherol in the lipid fraction of *J. excelsa* ripe galbuli from lacations 1 and 3, and its concentration varied depending on the location and ripening stage, which may also be a function of the environmental conditions [49,50]. Additionally, the content of *α*-tocotrienol decreased during the ripening of the galbuli (from 42.5% to 37.6% in the sample from location 2; from 66.2% to 6.1% in galbuli from location 1. Tocopherol composition of *J. excelsa* galbuli was completely different from that of sunflower, melon, pumpkin, and soybean oils as reported in the literature [51].

The secondary metabolites such as flavonoids and polyphenolic acids play a defensive role in plants. The extracts obtained from them are used in folk medicine or pharmaceutical and cosmetic products. There are reports on the chemical composition of *J. excelsa* galbuli essential oil [12,13]; however, there are no data on the phytochemical composition of extracts in different ripening stages from *J. excelsa* galbuli, especially in Bulgaria.

The antioxidant activity and phenolic content of *J. excelsa* essential oils and extracts have been studied [17,21]; however, information about a possible correlation between variables is limited. The values in this study were lower than those presented by Lesjak et al. [21] on phenolic content (94.7 mg GAE/g dw) and total flavonoids (30.5 QE/g dw) of the *J. excelsa* galbuli extracts from the location of island Golem Grad. The same authors reported noteworthy antioxidant activity determined by DPPH (5.3 µg/mL) and FRAP (86.7 mg of ascorbic acid equivalents/g of dw) of the *J. excelsa* galbuli extracts. Bakkour et al. [52] studied the antioxidant activity of the essential oil from unripe and ripe *J. excelsa* galbuli from Lebanon and revealed limited antioxidant activities for both of the two samples. Höferl et al. [20] studied the in vitro antioxidant activity of common juniper berry (*J. communis* L.) essential oil by DPPH scavenging in order to determine the ability of commercial juniper berry oil components to act as hydrogen atom donors. The authors reported that the oil had a weak DPPH radical reduction with an IC_50_ value of 34.8 mg/mL (*R*^2^ = 0.99). On the other hand, the essential oil showed a significant inhibitory effect of ABTS radicals (IC_50_ = 10.96 µg/mL, *R*^2^ = 0.901). El-Achi et al. [13] determined high phenolic content (17.9 mg/g of extract) and total flavonoid content (3.8 mg/g of extract) that are related to the strong scavenging activity with an IC_50_ = 48.9 µg/mL in Lebanese *J. excelsa* galbuli extract. Consistent with our findings, a correlation generally was observed between total phenol amounts of the ethanol extracts and their antioxidant activities measured by DPPH radical scavenging activity and FRAP assays [53]. A number of methods are used in order to study the antioxidant action of plants and the content of phenolic compounds and flavonoids. The differences between the results in this study and the literature reports are probably due to the application of the different extraction methods and methodology for the detection of phenols. Free radicals can be generated from metabolic pathways in the cell, as well as from external sources such as food, drugs, and environmental pollution. They can cause damage to various tissues and macromolecules in the body. The synthetic food additives and commercial antioxidants have been criticized, mainly for their possible toxic effects. This creates a growing interest in the antioxidant activity of natural compounds. Therefore, the *J. excelsa* extracts could be an alternative to the synthetic compounds used in the food and pharmaceutical industries.

The synthesis and accumulation of plant secondary metabolites, such as phenols, depends on environmental factors, including latitude and altitude. Location 3 is situated in the second-highest mountain in Bulgaria, Pirin, which may have contributed to the highest levels of phenolics in comparison with the other two locations. In addition, Martz et al. [54] explained the increase in soluble phenolic and terpenoid composition of juniper needles when they are collected from the northern hemisphere and with increasing altitude. A similar tendency for the domination of total phenols and flavonoids in the unripe fruits from other *Juniperus* species, including *J. communis*, *J. excelsa*, *J. foetidissima*, *J. oxycedrus*, and *J. sabina*, was previously reported [53]. The total content of phenols in *J. excelsa* unripe and ripe galbuli varied between 12 and 0.9 GAE/g dw, while total flavonoids were 0.5–4.3 QE/g dw. This is consistent with the reported facts for different juniper varieties that a significant difference (*p* < 0.05) in TPC and TFC values was observed between different varieties of juniper berries [43,53,55].

The antioxidant activity of *J. excelsa* unripe and ripe galbuli was evaluated by four antioxidant methods, based on different mechanisms. Better results were demonstrated by the method based on the electron transfer mechanism, the CUPRAC assay (Table 6). The 70% ethanol extract of unripe *J. excelsa* galbuli from location 3 also showed the highest antioxidant potential by copper reduction assay—473.9 mM TE/g dw. Antioxidant capacity can vary and depends on the sample and the nature and type of solvent extraction [56]. In general, it was reported that ethanol extract of *J. communis* showed better scavenging activity in the FRAP assay among five different *Juniperus species*, including *J. communis*, *J. excelsa*, *J. foetidissima*, *J. oxycedrus*, and *J. sabina* [53].

Total flavonoids slightly correlated with the antioxidant methods FRAP, ABTS, and DPPH in 95% ethanol extracts of *J. excelsa*. A significant correlation between the number of total phenols and the antioxidant activity by the FRAP method was found, while in the 70% ethanol extracts, there was a high correlation between the total phenols and the DPPH, ABTS, and CUPRAC methods. Therefore, the radical scavenging activity determined by the DPPH and ABTS methods was mostly influenced by total phenols, followed by the total amount of flavonoids. Among the metal-reducing methods, total phenols correlated with the CUPRAC method, while total flavonoids with the FRAP method. Overall, for all ethanol extracts, the most significant correlation between the total phenols and the DPPH method was observed (*R*^2^ = 0.92), and a negative correlation was found between total flavonoids and antioxidant activity. In general, the highest correlation was found between total phenols and all antioxidant methods in 70% ethanol extracts from the unripe galbuli.

## 4. Materials and Methods

### 4.1. Plant Materials

*Juniperus excelsa* M. Bieb. galbuli were collected in January, 2020 from the two protected areas: (1) Location 1—the reserve “Izgoryaloto Gyune”, above the town of Krichim, Bulgaria (42°01′40″ N, 24°28′09″ E, 367 masl), part of the Rodopi Mountain; (2) the reserve “Tisata” situated near the town of Kresna, Bulgaria (41°74′14″ N, 23°15′54″ E, 66 masl). The protected area “Tisata” consists of two sections—east and west—located in the Maleshevska Planina (Location 2) and Pirin Mountain (Location 3), respectively. Public access and commercial activities such as grazing are prohibited in the “Tisata” protected area and in reserve “Izgoryaloto Gyune” in order to preserve the natural diversity of these areas. A sampling permit was obtained from the Bulgarian Ministry of the Environment (№ 736/12.03.2018, issued to Tzenka Radoukova and Valtcho D. Zheljazkov). Voucher specimens of these species were deposited at the Herbarium of the Agricultural University, Plovdiv, Bulgaria (SOA) [57].

The galbuli were separated and placed in paper sacks and stored in a cool, dry, well-ventilated, and dark cabinet. The harvest involved a random sampling from 20 trees. The species were identified by Dr. Ivanka Semerdjieva and Tzenka Radoukova.

Prior to extraction, the galbuli were finely ground with a laboratory mill (Clatronic KSW 3307 Grinder, SC MELA-ROX COM SRL, Bistrita, Romania). The biologically active substances in the samples were analyzed, and the values were represented based on the absolute dry weight.

### 4.2. Moisture Content

The moisture of the galbuli was determined by drying up to the constant weight at 105 °C [58].

### 4.3. Protein Content

The total protein content was analyzed according to the method of AOAC 976.06 [59] with a UDK 152 Kjeldahl System (Velp Scientiffica, Usmate, Italy). The samples of 1.0 g each were mineralized in 15 mL concentrated H_2_SO_4_ and catalysts: anhydrous K_2_SO_4_ and CuSO_4_. The process was run at 420 °C for 60 min. With this method, 40% NaOH was used to produce an alkaline distillation medium and 4% H_3_BO_3_ in order to collect the distilled ammonia. The titrations were carried out with a standard HCl (0.2 N) solution.

#### Amino Acid Composition

The protein was hydrolyzed to free amino acids as 300 mg galbuli were placed in a glass ampule with a 5 mL 6N HCl solution. The ampule was thoroughly sealed and left in a drying chamber at 105 °C for 24 h. The ampule content was then transferred to a crystallizer and dried in a vacuum chamber at 40–50 °C. After evaporation of the water, the residue was fully diluted in 10 mL 20 mM HCl. The solution was filtered through a paper filter and 20 µL of the collected filtrate was derivatized with an AccQ-Fluor kit (WATO52880, Waters Corporation, Milford, MA, USA). Initially, 60 µL AccQ-Fluor borate buffer was added to the filtrate and homogenized. Then, 20 µL AccQ-Fluor reagent was added, and the sample was homogenized again for 30 s. Before injection, the solution was heated in a water bath at 55 °C. The resulting AccQ-Fluor amino acid derivatives were separated by an ELITE LaChrome high-performance liquid chromatography (Hitachi) equipped with a diode array detector and a reversed phase column C18 AccQ-Tag (3.9 × 150 mm) operating at 37 °C. The volume of the injected sample was 20 µL, and the elution was made with a gradient system of two mobile phases: A—buffer (WATO52890, Waters Corporation, Milford, MA, USA); B—60% acetonitrile. Amino acids were detected at 254 nm.

### 4.4. Total Chlorophylls and Carotenoid Content

For analysis of chlorophyll a, chlorophyll b, total chlorophylls, and the total carotenoids [60,61] in 95% ethanol extracts obtained from the fresh unripe and ripe galbuli, the absorbance was measured at three wavelengths—664, 648, and 470 nm. The amount of these pigments was calculated in µg/mL, according to the Formulas (1)–(4) of Lichtentaler and Wellburn [62].

Chlorophyll a (Ca) = 13.36A_664.2_ − 5.19A_648.6_ (1)

Chlorophyll b (Cb) = 27.43A_648.6_ − 8.12A_664.2_ (2)

Total Chlorophyll (a + b) = 5.24A_664.2_ + 22.24A_648.6_ (3)

Total carotenoids = [1000A_470_ − 2.13Ca − 97.64Cb]/209 (4)

### 4.5. Isolation of Essential Oil

The air-dried galbuli (50 g) were cut to a size of 0.5 cm. The essential oil was isolated by hydrodistillation (ratio galbuli:water = 1:10) for 3 h in a Clevenger-type laboratory glass apparatus of the British Pharmacopoeia. The oil obtained was dried over anhydrous sodium sulfate and stored in tightly closed dark vials at 4 °C until analysis.

### 4.6. Isolation of Lipid Fraction

The lipid fraction was extracted from ground galbuli (12 g) using *n*-hexane in a Soxhlet apparatus for 8 h. The solvent was partially removed by a rotary vacuum evaporator, the residue was transferred in a preweighed glass vessel, and the rest of the solvent was removed under a stream of nitrogen to a constant weight to determine the oil content [63].

#### 4.6.1. Fatty Acid Composition

The fatty acid composition of triacylglycerols was determined by gas chromatography (GC) [63]. Fatty acid methyl esters (FAMEs) were prepared by pre-esterification of the triacylglycerols with sulfuric acid in methanol [64]. The determination of FAMEs was performed on HP5890 gas chromatograph equipped with a 75 m × 0.18 mm × 25 μm (film thickness) capillary Supelco column and a flame ionization detector. The column temperature was programmed from 140 °C (held 5 min), at 4 °C/min to 240 °C (held 3 min); the injector and detector temperatures were set at 250 °C. Identification was performed by comparison of the retention times with those of a standard mixture of FAME (Supelco, USA 37 comp. FAME mix) subjected to GC under identical experimental conditions. The limit of detection in GC was 0.05%.

#### 4.6.2. Sterols

Unsaponifiables were determined after saponification of the lipid fraction and extraction with *n*-hexane [65]. Quantification of sterols was carried out spectrophotometrically (at 597 nm), after the isolation of sterols from other unsaponifiable matter by TLC [66]. For the determination of total sterols, an analytical curve was constructed by using a standard solution of *β*-sitosterol—the concentration ranged from 0 to 3000 μg/mL. The linear regression coefficient (*R*^2^) was 0.9985, the limit of detection was calculated to be 95 μg/mL, and the limit of quantification was 315 μg/mL.

Sterol composition was determined on a HP 5890 gas chromatograph equipped with 25 m × 0.25 mm DB—5 capillary column and flame ionization detector. The temperature gradient was from 90 (held 3 min) up to 290 °C at a rate of change of 15 °C/min and then up to 310 °C at a rate of 4 °C/min (held 10 min); detector temperature—320 °C; injector temperature—300 °C hydrogen as a carrier gas. Identification was confirmed by the comparison of retention times with those of a standard mixture of sterols containing cholesterol (stabilized, purity 95%, Acros Organics, Morris Plains, NJ, USA), stigmasterol (Sigma-Aldrich, purity 95%, St. Louis, MO, USA), and β-sitosterol (with ca 10% campesterol, ca 75% *β*-sitosterol, Acros Organics, Morris Plains, NJ, USA) [67]. The limit of detection in GC was 0.05%.

#### 4.6.3. Tocopherols

Tocopherols were determined directly in the oil by high-performance liquid chromatography on a Merck-Hitachi (Merck, Darmstadt, Germany) instrument equipped with 250 × 4 mm Nucleosil Si 50–5 column and fluorescent detector Merck-Hitachi F1000. The operating conditions were a mobile phase of *n*-hexane:dioxane, 96:4 (*v/v*) and flow rate 1 mL/min, excitation 295 nm, emission 330 nm. An amount of 20 μL 2% solution of crude oil in *n*-hexane was injected. Tocopherols were identified by comparing the retention times with those of authentic individual ones. The tocopherol content was calculated on the base of tocopherol peak areas in the sample vs. the tocopherol peak area of the standard tocopherol solution [68,69]. The limit of detection in HPLC was 0.05%.

### 4.7. Composition and Antioxidant Activity of Extracts of J. excelsa Galbuli

#### 4.7.1. Extraction Procedure

The ultrasound-assisted extraction of biologically active substances from galbuli was performed in an ultrasonic bath SIEL UST 5.7–150 (Gabrovo, Bulgaria) with a frequency of 35 kHz and 300 W. The extraction procedure was performed with two solvents with different polarity 95% (*v/v*) and 70% aqueous ethanol in a solvent to solid ratio (1:5 *v/w*). The plant materials were weighed in a 50 mL centrifuge tube with a screw cap, and then 20 mL solvent was added to the sample. The tubes were placed in the ultrasonic bath at 45 °C for 20 min. The ultrasound-assisted extraction was performed in triplicate. Each extract was filtered, and the combined extracts were used for further analysis.

#### 4.7.2. Determination of Total Phenolic Content (TPC)

The total phenolic content (TPC) was determined using the Folin–Ciocalteu reagent [70]. Each galbuli extract (0.2 mL) was mixed with 1 mL Folin–Ciocalteu reagent (diluted five times) and 0.8 mL 7.5% Na_2_CO_3_. After 20 min at room temperature (25 °C) in darkness, the absorption was measured at 765 nm against a blank sample. The TPC was expressed as mg gallic acid equivalent (GAE) per g dry weight (dw). The TPC was expressed as mg gallic acid equivalent (GAE) per g dry weight (dw).

#### 4.7.3. Determination of Total Flavonoid Content (TFC)

The total flavonoid content was analyzed by Al(NO_3_)_3_ reagent [71]. The extract (1 mL) was mixed with 0.1 mL 10% aluminum nitrate, 0.1 mL 1M potassium acetate, and 3.8 mL 95% ethanol. After 40 min, the absorbance was measured at 415 nm against a blank as the above-mentioned procedure, prepared without 1 M potassium acetate. The results were expressed as mg quercetin equivalents (QE) per g dw.

#### 4.7.4. Antioxidant Activity

##### 2,2-diphenyl-1-picryl-hydrazyl-hydrate (DPPH) Radical Scavenging Activity

The DPPH radical scavenging activities of galbuli were evaluated as the plant extract (0.15 mL) was added to 2.85 mL freshly prepared 0.1 mM DPPH solution in methanol. The samples were incubated for 15 min at 37 °C in darkness. The reduction in the absorbance at 517 nm was measured by a spectrophotometer in comparison to the blank containing methanol [72]. Radical scavenging activity of galbuli was expressed as mM Trolox^®^ equivalent (TE) per g/dw.

##### 2,2′-Azino-bis(3-Ethylbenzothiazoline-6-Sulfonic Acid) (ABTS) Assay

The radical action 2,2′-azinobis (3-ethylbenzothiazoline-6-sulfonic acid) (ABTS•+) was generated by mixing an aliquot part of 7.0 mM 2,2′azinobis (3)ethylbenzthiazoline-6-sulfonic acid (ABTS, Sigma) in d. H_2_O. with 2.45 mM potassium persulfate in d. H_2_O. The reaction was performed for 16 h at ambient temperature in darkness. Before analyses, 2.0 mL of generating ABTS•+ solution was diluted with methanol at proportions 1:30 (*v/v*), to the final absorbance of the working solution of about 1.0 ÷ 1.1 at a wavelength of 734 nm. ABTS + solution (2.85 mL) was added to 0.15 mL extracts. After 15 min at 37 °C in darkness, the absorbance was measured at 734 nm against methanol [73]. The results were expressed as mM TE per g/dw.

##### Ferric Reducing Antioxidant Power Assay (FRAP)

The FRAP method was performed as previously described [74]. Three mL freshly prepared FRAP reagent (consisting of 10 parts 0.3 M acetate buffer (pH 3.6), 1 part 10 mM 2,4,6-tripyridyl-s-triazine (TPTZ) in 40 mM HCl, and 1 part 20 mM FeCl_3_·6H_2_O in d. H_2_O) were mixed with 0.1 mL of the plant extract. After 10 min at 37 °C in darkness, the absorbance was measured at 593 nm against a blank prepared with the used solvent for extraction. The results were expressed as mM TE per g/dw.

##### Cupric Ion Reducing Antioxidant Capacity (CUPRAC)

The CUPRAC assay was used [75]. One mL CuCl_2_ × 2H_2_O, 1 mL neocuproine (7.5 mL in methanol), 0.1 M ammonium acetate buffer (1 mL) and 1 mL d. H_2_O were mixed with 0.1 mL extracts. The reaction was performed for 20 min at 50 °C in darkness. After cooling to room temperature (25 °C), the absorbance of samples was measured at 450 nm against a blank. Trolox^®^ was used as a standard and the total antioxidant activity was expressed as mM TE per g dw.

### 4.8. Statistical Analysis

The measurements were performed in triplicate, and the results were presented as the mean value of the individual measurements with the corresponding standard deviation (SD), using Microsoft Excel. A significantly different at *p* ≤ 0.05 was measured by Tukey’s multiple range test, using ANOVA.

## 5. Conclusions

To the best of our knowledge, this is the first extensive report on the phytochemical composition of *J. excelsa* unripe and ripe galbuli from natural habitats of species in Bulgaria. There is no information on the biochemical composition of *J. excelsa* galbuli in other countries where the species is widespread. The study revealed that the proximate composition of *J. excelsa* galbuli is strongly influenced by the stage of maturity and environmental factors. The focus on antioxidants naturally occurred in plants is related to their application aimed at the prevention of oxidative damage to biological systems by reactive oxygen species. The findings in this study confirm the genotype and environment effect of the chemical profile of *J. excelsa* galbuli.

## Figures and Tables

**Table 1:** Proximate composition of *J. excelsa* unripe and ripe galbuli from the three collection sites in Bulgaria (mean ± SD).

Indicators	Location 1		Location 2		Location 3	
	Unripe	Ripe	Unripe	Ripe	Unripe	Ripe
Moisture, %	37.4 ± 3.6	36.9 ± 3.5	37.7 ± 3.5	29.6 ± 2.8	31.9 ± 3.0	32.0 ± 3.0
Protein, %	14.4 ± 1.4	13.6 ± 1.3	16.4 ± 1.5	14.9 ± 1.4	13.9 ± 1.3	15.4 ± 1.5
Chlorophyll a, μg/g dw	121.2 ± 12.0	128.6 ± 12.0	126.4 ± 11.5	117.6 ± 10.0	94.2 ± 9.2	193.1 ± 18.0
Chlorophyll b, μg/g dw	64.2 ± 6.3	58.4 ± 5.2	83.5 ± 8.0	58.7 ± 5.5	51.5 ± 5.0	80.3 ± 7.8
Total Chlorophyll, μg/g dw	185.4 ± 18.0	187.1 ± 17.2	209.9 ± 19.0	176.3 ± 17.0	145.6 ± 13.6	273.4 ± 25.0
Total Carotenoid, μg/g dw	49.5 ± 4.5	50.3 ± 5.0	47.0 ± 4.5	41.7 ± 4.0	46.5 ± 4.5	50.4 ± 5.0
Essential oil yield, %	1.9 ± 0.2	5.1 ± 0.5	1.9 ± 0.2	2.6 ± 0.2	- *	2.5 ± 0.2
Lipid fraction, %	6.6 ± 0.6	9.1 ± 0.9	7.0 ± 0.7	5.5 ± 0.5	-	4.5 ± 0.4

* not analyzed due to insufficient sample quantity; Location 1—Krichim, Reserve “Izgorjaloto Gyune”; Location 2—Reserve “Tisata”/Malashevska Mountain; Location 3—Reserve “Tisata”/Eastern/Pirin Mountain.

**Table 2:** Fatty acid composition of the lipid fraction of *J. excelsa* unripe and ripe galbuli from the three collection sites in Bulgaria (mean ± SD), %.

Fatty Acid		Location 1		Location 2		Location 3 **
		Unripe	Ripe	Unripe	Ripe	Ripe
Capric acid	C _10:0_	* -	0.4 ± 0.0	* -	0.5 ± 0.0	0.4 ± 0.0
Lauric acid	C _12:0_	0.7 ± 0.0	0.5 ± 0.0	1.3 ± 0.9	0.8 ± 0.0	0.7 ± 0.0
Tridecanoic acid	C _13:0_	0.2 ± 0.0	0.2 ± 0.0	* -	0.6 ± 0.0	0.6 ± 0.0
Myristic acid	C _14:0_	1.3 ± 0.1	0.6 ± 0.0	2.5 ± 0.1	1.6 ± 0.0	1.2 ± 0.0
Myristoleic acid	C _14:1_	0.9 ± 0.0	0.4 ± 0.0	2.4 ± 1.2	1.4 ± 0.0	1.3 ± 0.0
Pentadecenoic acid	C _15:1_	0.3 ± 0.0	0.2 ± 0.0	0.8 ± 0.0	0.3 ± 0.0	0.3 ± 0.0
Palmitic acid	C _16:0_	31.8 ± 2.6	29.9 ± 3.0	41.3 ± 2.7	31.2 ± 2.4	32.2 ± 2.4
Palmitoleic acid	C _16:1_	0.3 ± 0.0	* -	* -	0.8 ± 0.0	1.1 ± 0.0
Margaric acid	C _17:0_	0.3 ± 0.0	0.2 ± 0.0	0.3 ± 0.0	0.3 ± 0.0	0.2 ± 0.0
Heptadecenoic acid	C _17:1_	0.3 ± 0.0	0.4 ± 0.0	0.6 ± 0.0	0.4 ± 0.0	0.5 ± 0.0
Stearic acid	C _18:0_	4.5 ± 0.1	8.4 ± 1.0	4.7 ± 1.0	6.8 ± 1.0	6.7 ± 0.0
Oleic acid	C _18:1_	19.4 ± 1.1	22.8 ± 2.5	8.4 ± 1.0	30.0 ± 2.3	32.6 ± 0.0
trans	C _18:1_	* -	* -	* -	0.3 ± 0.0	* -
Linoleic acid	C _18:2_	26.2 ± 2.2	23.9 ± 2.2	17.5 ± 2.7	15.2 ± 1.1	13.2 ± 1.1
Linolenic acid	C _18:3_	1.9 ± 0.1	4.8 ± 0.0	1.3 ± 0.0	2.5 ± 0.1	1.9 ± 0.1
Heneicosanoic acid	C _21:0_	0.6 ± 0.0	0.4 ± 0.0	0.9 ± 0.0	0.3 ± 0.0	0.4 ± 0.0
Arachidic acid	C _20:0_	* -	0.2 ± 0.0	0.3 ± 0.0	* -	0.2 ± 0.0
Eicosadienoic acid	C _20:2_	0.2 ± 0.0	0.6 ± 0.0	2.3 ± 1.0	0.9 ± 0.0	1.6 ± 0.0
Eicosatrienoic acid	C _20:3_	8.8 ± 0.6	3.9 ± 0.1	7.1 ± 0.0	2.1 ± 0.1	1.7 ± 0.0
Eicosapentaenoic acid	C _20:5_	* -	* -	* -	0.3 ± 0.0	* -
Erucic acid	C _22:1_	* -	* -	0.7 ± 0.0	* -	* -
Docosadienoic acid	C _22:2_	0.3 ± 0.0	0.2 ± 0.0	0.5 ± 0.0	0.3 ± 0.0	0.2 ± 0.0
Lignoceric acid	C _24:0_	2.0 ± 0.0	1.8 ± 0.1	6.4 ± 1.0	2.7 ± 0.1	2.7 ± 0.1
Nervonic acid	C _24:1_	* -	0.2 ± 0.00	0.7 ± 0.00	0.7 ± 0.00	0.3 ± 0.00

* not identified; ** unripe galbuli not analyzed due to insufficient sample quantity; Location 1—Krichim, Reserve ”Izgorjaloto Gyune”; Location 2—Reserve “Tisata”/Malashevska Mountain; Location 3—Reserve “Tisata”/Eastern/Pirin Mountain.

**Table 3:** Biologically active substances in the lipid fraction of *J. excelsa* unripe and ripe galbuli from the three collection sites in Bulgaria (mean ± SD).

Biologically Active Substances	Location 1		Location 2		Location 3 *
	Unripe	Ripe	Unripe	Ripe	Ripe
Unsaponifiable matter, %	9.8 ± 0.1	5.5 ± 0.1	11.6 ± 0.1	11.4 ± 0.2	13.5 ± 0.3
Sterols, %	0.2 ± 0.0	0.1 ± 0.0	0.2 ± 0.04	0.3 ± 0.0	0.3 ± 0.0
Tocopherols, mg/kg	625 ± 10.0	1894 ± 21.0	721 ± 12.02	1457 ± 20.1	1476 ± 15.0

* unripe galbuli not analyzed due to insufficient sample quantity; Location 1—Krichim, Reserve “Izgorjaloto Gyune”; Location 2—Reserve “Tisata”/Malashevska Mountain; Location 3—Reserve “Tisata”/Eastern/Pirin Mountain.

**Table 4:** Sterol and tocopherol composition of the lipid fraction of *J. excelsa* unripe and ripe galbuli from the three collection sites in Bulgaria (mean ± SD), %.

Sterol and Tocopherol Composition	Location 1		Location 2		Location 3 **
	Unripe	Ripe	Unripe	Ripe	Ripe
Sterols					
Cholesterol	0.2 ± 0.0	0.5 ± 0.0	0.5 ± 0.0	- *	0.7 ± 0.0
Brasicasterol	5.5 ± 0.2	6.4 ± 0.1	7.3 ± 0.3	5.9 ± 0.2	6.7 ± 0.2
Campesterol	13.9 ± 0.3	- *	9.9 ± 0.3	9.2 ± 0.2	26.5 ± 0.5
Stigmasterol	8.9 ± 0.2	- *	3.3 ± 0.2	4.6 ± 0.3	- *
*β*-Sitosterol	70.3 ± 0.6	91.7 ± 0.6	76.7 ± 0.6	71.9 ± 0.6	64.8 ± 0.4
Δ^5^-Avenasterol	0.4 ± 0.0	0.8 ± 0.1	2.1 ± 0.1	- *	0.5 ± 0.1
Δ^7^-Stigmasterol	0.8 ± 0.1	0.6 ± 0.01	0.2 ± 0.0	8.4 ± 0.2	0.8 ± 0.1
Tocopherols					
*α*-Tocopherol	33.8 ± 0.3	85.5 ± 0.6	57.5 ± 0.5	19.0 ± 0.3	88.5 ± 0.6
*α*-Tocotrienol	66.2 ± 0.45	6.1 ± 0.2	42.5 ± 0.5	37.6 ± 0.3	11.5 ± 0.3
*β*-Tocopherol	- *	- *	- *	17.2 ± 0.2	- *
*γ*-Tocopherol	- *	8.4 ± 0.4	- *	26.2 ± 0.2	- *

* not identified; ** unripe galbuli not analyzed due to insufficient sample quantity; Location 1—Krichim, Reserve ”Izgorjaloto Gyune”; Location 2—Reserve “Tisata”/Malashevska Mountain; Location 3—Reserve “Tisata”/Eastern/Pirin Mountain.

**Table 5:** Amino acid composition of the protein fraction of *J. excelsa* unripe and ripe galbuli from the three collection sites in Bulgaria (mean ± SD), mg/g.

Amino Acid	Location 1		Location 2		Location 3	
	Unripe	Ripe	Unripe	Ripe	Unripe	Ripe
Asparagine	8.6 ± 0.1	7.2 ± 0.1	5.9 ± 0.0	4.7 ± 0.1	3.4 ± 0.2	6.9 ± 0.1
Serine	1.9 ± 0.0	1.8 ± 0.0	4.0 ± 0.1	2.4 ± 0.0	0.9 ± 0.0	2.5 ± 0.0
Glutamic acid	4.0 ± 0.0	2.9 ± 0.0	6.7 ± 0.1	3.9 ± 0.0	2.8 ± 0.0	3.8 ± 0.0
Glycine	1.0 ± 0.0	0.7 ± 0.0	1.2 ± 0.0	1.0 ± 0.0	0.8 ± 0.0	1.0 ± 0.1
Histidine	6.5 ± 0.1	5.5 ± 0.1	7.6 ± 0.1	8.0 ± 0.1	6.1 ± 0.1	6.8 ± 0.1
Arginine	3.2 ± 0.1	2.7 ± 0.1	3.4 ± 0.1	3.3 ± 0.0	2.5 ± 0.1	3.2 ± 0.1
Thryptophan	2.4 ± 0.1	2.2 ± 0.0	3.1 ± 0.1	3.5 ± 0.1	2.2 ± 0.1	2.8 ± 0.1
Alanine	5.1 ± 0.1	4.6 ± 0.1	6.3 ± 0.1	7.1 ± 0.1	4.8 ± 0.1	5.4 ± 0.1
Proline	2.2 ± 0.1	5.4 ± 0.1	2.9 ± 0.1	3.5 ± 0.1	2.1 ± 0.1	2.7 ± 0.1
Cysteine	trace *	trace	0.1 ± 0.0	0.1 ± 0.0	trace	trace
Tyrosine	1.2 ± 0.0	1.7 ± 0.0	1.3 ± 0.0	2.2 ± 0.0	1.2 ± 0.0	1.9 ± 0.0
Valine	3.1 ± 0.0	2.7 ± 0.0	3.8 ± 0.1	4.2 ± 0.1	2.9 ± 0.1	4.2 ± 0.1
Methionine	0.2 ± 0.0	0.2 ± 0.0	0.30 ± 0.0	0.3 ± 0.0	0.3 ± 0.0	0.9 ± 0.0
Lysine	4.4 ± 0.1	4.0 ± 0.1	5.5 ± 0.1	5.7 ± 0.1	4.8 ± 0.1	6.1 ± 0.1
Isoleucine	3.2 ± 0.1	2.8 ± 0.0	3.9 ± 0.1	4.4 ± 0.1	2.9 ± 0.1	3.4 ± 0.1
Leucine	0.5 ± 0.0	0.4 ± 0.0	0.6 ± 0.0	0.7 ± 0.0	0.6 ± 0.0	0.5 ± 0.0
Phenylalanine	2.9 ± 0.0	2.5 ± 0.0	3.3 ± 0.1	4.0 ± 0.1	2.4 ± 0.0	5.3 ± 0.1

* trace—< 0.05 mg/g; Location 1—Krichim, Reserve ”Izgorjaloto Gyune”; Location 2—Reserve “Tisata”/Malashevska Mountain; Location 3—Reserve “Tisata”/Eastern/Pirin Mountain.

**Table 6:** Total phenolic content (mg GAE/g dw), flavonoid content (mg QE/g dw), and antioxidant activity (mM TE/g dw) of *J. excelsa* unripe and ripe galbuli from three collection sites in Bulgaria (mean ± SD). For each column, within the same series (ripe or unripe) different lower case letters indicate significantly different at *p* ≤ 0.05 as measured by Tukey’s multiple range test. *(*p* < 0.05) and **(*p* < 0.01) indicate significant differences between ripe and unripe (Student’s *t*-test).

Total Phenolic and Flavonoid Content and Antioxidant Activity	Location 1		Location 2		Location 3	
	Unripe	Ripe	Unripe	Ripe	Unripe	Ripe
solvent 95% ethanol						
TPC, mg GAE ^1^/g dw ^2^	4.0 ± 0.5 ^b,^*	3.1 ± 0.1 ^b,c,^**	4.2 ± 0.1 ^ns^	2.06 ± 0.2 ^d,^**	4.28 ± 0.4 ^a,ns^	2.0 ± 0.1 ^e,^**
TFC, mg QE ^3^/g dw	1.8 ± 0.0 ^b,^**	2.1 ± 0.1 ^c,^**	1.7 ± 0.3 ^b^^,^*	0.5 ± 0.0 ^ns^	4.3 ± 0.0 ^a,^**	3.1 ± 0.0 ^d,^**
DPPH, mM TE/g dw	23.2 ± 1.1 ^d,^**	6.9 ± 1.0.1 ^c,^**	24.8 ± 0.1 **	11.5 ± 1.1 ^e,^**	34.8 ± 0.0 ^a,^**	10.0 ± 0.0 ^e,f,^**
ABTS, mM TE/g dw	66.3 ± 0.8 **	26.7 ± 1.7 ^b,^**	68.1 ± 1.4 ^a,c,ns^	35.0 ± 2.3 ^d,^**	90.4 ± 0.3 ^e,^**	32.3 ± 2.6 ^d,f,ns^
FRAP, mM TE/g dw	40.3 ± 1.3 **	14.4 ± 2.7 ^b,^**	37.9 ± 2.6 ^a,c,ns^	18.5 ± 1.9 ^b,d,ns^	46.7 ± 1.0 ^e,^**	15.6 ± 2.9 ^d,f,ns^
CUPRAC, mM TE/g dw	71.0 ± 0.5 ^a,^**	47.1 ± 0.7 ^b,^**	72.5 ± 5.6 ^c,ns^	50.5 ± 0.1 ^ns^	101.0 ± 1.0 ^d,^**	36.0 ± 3.8 ^b,^**
solvent 70% ethanol						
TPC, mg GAE/g dw	2.3 ± 0.1 ^b,^**	3.0 ± 0.2 ^ns^	0.9 ± 0.0 ^c,^**	2.8 ± 0.1 ^ns^	12.3 ± 0.6 ^a,^**	2.1 ± 0.2 ^ns^
TFC, mg QE/g dw	0.5 ± 0.0 ^a,^**	1.5 ± 0.0 ^b,^**	0.8 ± 0.0 ^c,^**	0.4 ± 0.1 ^d,^**	1.66 ± 0.0 ^e,^**	0.6 ± 0.0 ^f,^**
DPPH, mM TE/g dw	69.8 ± 3.5 ^a,^**	29.5 ± 1.2 ^b,^**	38.0 ± 2.3 ^b,c,ns^	20.3 ± 5.1 ^b,d,ns^	372.9 ± 6.5 ^a,^**	21.7 ± 6.1 ^d,f,ns^
ABTS, mM TE/g dw	119.0 ± 5.6 ^ns^	89.1 ± 6.7 ^ns^	120.2 ± 5.1 ^ns^	6996 ± 10.2 ^b,^**	297.6 ± 7.3 ^a,^**	37.8 ± 0.4 ^c,^**
FRAP, mM TE/g dw	71.8 ± 0.4 ^b,^*	60.1 ± 0.9 ^c,^**	78.9 ± 1.1 ^d,^**	28.0 ± 0.7 ^e,^**	184.9 ± 1.7 ^a,^**	22.4 ± 1.2 **
CUPRAC, mM TE/g dw	148.0 ± 1.0 ^b,^**	5.5 ± 0.0 ^c,^**	131.1 ± 1.2 ^d,^**	64.0 ± 9.0 ^e,^**	473.9 ± 2.0 ^a,^**	49.3 ± 2.0 ^f,^**

^1^ GAE—gallic acid equivalent; ^2^ dw—dry weight; ^3^ QE—quercetin equivalent; TPC and TFC: total phenolic content and total flavonoid content, DPPH—2,2-diphenyl-1-picryl-hydrazyl-hydrate, ABTS—2,2′-Azino-bis(3-Ethylbenzothiazoline-6-Sulfonic Acid), FRAP—ferric reducing antioxidant power assay, CUPRAC—cupric ion reducing antioxidant capacity, ns—not significant, *(*p* < 0.05) and **(*p* < 0.01), a,b,c,d,e,f letters indicate significantly different at *p* ≤ 0.05 as measured by Tukey’s multiple range test; Location 1—Krichim, Reserve ”Izgorjaloto Gyune”; Location 2—Reserve “Tisata”/Malashevska Mountain; Location 3—Reserve “Tisata”/Eastern/Pirin Mountain.

**Table 7:** Correlation (*r*^2^) between phenolic compounds and antioxidant activity of *J. excelsa* unripe and ripe galbuli from the different parts of Bulgaria.

	DPPH	ABTS	FRAP	CUPRAC
Correlation (*r*^2^) between phenolic compounds and antioxidant activity in all 95% ethanol extracts				
TPC *, mg GAE/g dw ***	0.8329	0.8573	0.8908	0.8610
TFC **, mg QE/g dw	0.4866	0.4778	0.3709	0.4644
Correlation (*r*^2^) between phenolic compounds and antioxidant activity in the 95% ethanol extracts from the unripe galbuli				
TPC, mg GAE/g dw	0.8030	0.7654	0.5121	0.7506
TFC, mg QE/g dw	0.9870	0.9949	0.9730	0.9969
Correlation (*r*^2^) between phenolic compounds and antioxidant activity in the 95% ethanol extracts in the ripe galbuli				
TPC, mg GAE/g dw	−0.9309	−0.9312	−0.6916	0.3392
TFC, mg QE/g dw	−0.4420	−0.4413	−0.7777	−0.9090
Correlation (*r*^2^) between phenolic compounds and antioxidant activity in all 70% ethanol extracts				
TPC, mg GAE/g dw	0.9718	0.9023	0.8753	0.9302
TFC, mg QE/g dw	0.6582	0.6810	0.7300	0.6261
Correlation (*r*^2^) between phenolic compounds and antioxidant activity in the 70% ethanol extracts from the unripe galbuli				
TPC, mg GAE/g dw	0.9996	0.9930	0.9858	0.9976
TFC, mg QE/g dw	0.9435	0.9699	0.9809	0.9567
Correlation (*r*^2^) between phenolic compounds and antioxidant activity in the 70% ethanol extracts of the ripe galbuli				
TPC, mg GAE/g dw	0.5606	0.3115	0.7675	−0.4732
TFC, mg QE/g dw	0.9996	−0.6355	0.9525	−0.9974
Correlation (*r*^2^) between phenolic compounds and antioxidant activity in both ethanol extracts				
TPC, mg GAE/g dw	0.9220	0.8346	0.8105	0.8859
TFC, mg QE/g dw	−0.0082	−0.0289	−0.0491	−0.0152

* TPC—total phenolic content, ** TFC—total flavonoids content, *** dw—dry weight, respectively.
